# Microsporidia infection impacts the host cell's cycle and reduces host cell apoptosis

**DOI:** 10.1371/journal.pone.0170183

**Published:** 2017-02-02

**Authors:** Raquel Martín-Hernández, Mariano Higes, Soledad Sagastume, Ángeles Juarranz, Joyce Dias-Almeida, Giles E. Budge, Aránzazu Meana, Neil Boonham

**Affiliations:** 1 Laboratorio de Patología Apícola, Centro de Investigación Apícola y Agroambiental, IRIAF, Consejería de Agricultura de la Junta de Comunidades de Castilla-La Mancha, Marchamalo, Spain; 2 Instituto de Recursos Humanos para la Ciencia y la Tecnología (INCRECYT-FEDER), Fundación Parque Científico y Tecnológico de Albacete, Albacete, Spain; 3 Departamento de Biología, Facultad de Ciencias, Universidad Autónoma de Madrid, Madrid, Spain; 4 Fera, Sand Hutton, York, United Kingdom; 5 Institute for Agri-Food Research and Innovation, Newcastle University, Newcastle upon Tyne, United Kingdom; 6 Departamento de Sanidad Animal, Facultad de Veterinaria, Universidad Complutense de Madrid, Madrid, Spain; Institute of Plant Physiology and Ecology Shanghai Institutes for Biological Sciences, CHINA

## Abstract

Intracellular parasites can alter the cellular machinery of host cells to create a safe haven for their survival. In this regard, microsporidia are obligate intracellular fungal parasites with extremely reduced genomes and hence, they are strongly dependent on their host for energy and resources. To date, there are few studies into host cell manipulation by microsporidia, most of which have focused on morphological aspects. The microsporidia *Nosema apis* and *Nosema ceranae* are worldwide parasites of honey bees, infecting their ventricular epithelial cells. In this work, quantitative gene expression and histology were studied to investigate how these two parasites manipulate their host’s cells at the molecular level. Both these microsporidia provoke infection-induced regulation of genes involved in apoptosis and the cell cycle. The up-regulation of *buffy* (which encodes a pro-survival protein) and *BIRC5* (belonging to the Inhibitor Apoptosis protein family) was observed after infection, shedding light on the pathways that these pathogens use to inhibit host cell apoptosis. Curiously, different routes related to cell cycle were modified after infection by each microsporidia. In the case of *N*. *apis*, *cyclin B1*, d*acapo* and *E2F2* were up-regulated, whereas only *cyclin E* was up-regulated by *N*. *ceranae*, in both cases promoting the G1/S phase transition. This is the first report describing molecular pathways related to parasite-host interactions that are probably intended to ensure the parasite’s survival within the cell.

## Introduction

Parasitism is a type of biological interaction between organisms of different species whereby the parasite benefits at the expense of the host. Host cells have developed a defense machinery to resist pathogen invasion and replication. In order to limit pathogen growth, these systems include mechanism such as the fusion of phagolysosomals, the production of reactive oxygen and reactive nitrogen intermediates, nutrient sequestration or cell suicide (apoptosis) in order to limit pathogen growth [1]. As a counterpoint, obligate parasites have to exploit their host to complete the processes critical for their survival. It is interesting how some intracellular parasites can reprogram their host’s cells to create a safe haven, utilizing the cellular machinery to acquire the necessary resources [2]. The implementation of these defence systems and the ability of successful pathogens to mitigate their effects are ultimately mediated by changes in both the levels and activities of key proteins.

Apoptosis is one mechanism the host uses to control pathogen dissemination, and it is a morphologically and biochemically distinct form of programmed cell death. Apoptosis plays a major role in the removal of damaged or unwanted cells during development, in maintaining tissue homeostasis and it is involved in the aging of multicellular organisms. It is also recognized as an important defense mechanism of defence against viral, bacterial and parasitic pathogens during innate and adaptive immunity [3]. However some intracellular parasites can modulate apoptosis by either inducing or inhibiting the process thereby allowing the parasite to successfully reproduce [3, 4, 5, 6, 7].

Intracellular pathogens may also have other effects on infected cells. For example, *Toxoplasma cruzi* induces broad modulations of the host’s cellular machinery, altering the expression of up to 353 murine genes. This modification provides insight into how the parasite survives, replicates, and persists in the infected host, and ultimately it defines the clinical outcome of infection [8]. In addition, the host cell’s proteome responds in a dramatic way to *Toxoplasma gondii* infection, causing changes in protein expression and modifications to key metabolic pathways, including glycolysis, lipid and sterol metabolism, mitosis, apoptosis, and structural-protein expression. These changes suggest that the parasite provokes global reprogramming of the cell’s metabolism, revealing a complex molecular relationship between the host and parasite [1].

Microsporidia are eukaryotes, obligate intracellular fungal parasites that infect a wide range of vertebrates and invertebrates, and cause economically important losses in animal species. Microsporidian genomics and cell biology are the consequence of an extreme reduction driven by an intimate relationship with the host cell. During their life cycle, they have an extracellular spore (called environmental spore) but apart from this form, all the remaining stages develop during the infection of the host, maintaining direct contact with the host throughout the life cycle, from entry to egress.

Although microsporidial infection has often been linked to immunocompromised individuals in vertebrate hosts, there is no such prerequisite for the infection of insects. Indeed, microsporidia can cause serious disease in economically relevant insects like silkworm and crop pollinators. In this regard, *Apis mellifera* bees are recognized as a model system to study social interactions, immunity and disease in social insects [9]. Specifically, honey bees infection by microsporidia has been associated with significant colony losses [10]. To date, two microsporidian species are known to infect *A*. *mellifera* worldwide: *Nosema apis* [11] and *N*. *ceranae* [12]. Both infect the epithelial cells of the honey bee ventriculus (digestive tract) but the pathogenic events in the target tissue have not been described in detail.

Until recently there were only few studies of how microsporidia manipulated host cells and most of these were focused on morphology. In the last few years, it has become clear that some microsporidia produce more specific changes to gene expression in the infected host than other pathogens [13]. Nowadays, this large group of organisms is described as highly exploitative intracellular parasites of the host cell environment [14]. In terms of honey bee microsporidia, previous studies have shown that *N*. *ceranae* reduces apoptosis in the bee ventriculi which is the target tissue [15, 16] and induced an effect on genes involved in the homeostasis and renewal of intestinal tissues [17]. However, how this parasite affects other processes in host’s cells that serve to protect them against infection remains unclear.

Here we explore how intracellular parasites manipulate their host cell environment at the molecular level by studying quantitative gene expression in tissues following infection of honey bees with the both *Nosema* species. Our aim was to study the modifications that infection by these parasites produces in the process of host cell cycle and in the apoptosis by determining the expression of genes involved in the pathways related with these events. In parallel, we used TUNEL (Terminal deoxynucleotide transferase mediated X-dUTP nick end labelling) to assess the rate of apoptosis in these infected tissues after induction.

## Materials and methods

### Experimental infection

Honey bees used in this work were collected from experimental colonies located at “Centro de Investigación Apícola y Agroambiental- IRIAF”. No permits for the use of bees were required and the field studies did not involve endangered or protected species. A frame with capped brood of *A*. *mellifera iberiensis* worker bees was taken from a *Nosema*-free colony and maintained in an incubator at 35°C until the bees emerged [18, 19]. Newly emerged worker bees were carefully removed from the frame, confined to cages and kept in a different incubator for five days at 33°C. Individual bees were infected as described previously [18], using 2 μl of water containing 100,000 *N*. *ceranae* or *N*. *apis* spores. Spores were Percoll-purified and confirmed as single species by PCR [20]. Uninfected control bees were fed with 2 μl of water alone. In total, 25 bees were included in each group (*N*. *apis*, *N*. *ceranae* and Control). Ten days after infection 15 bees per group were treated with 2 μl of 2% cycloheximide solution (Sigma C7698; 94% purity), as an apoptosis inducer by death receptors [21], while the remaining 10 bees for each group were not treated with cycloheximide. The full experimental design is shown in Table 1. In this way, a total of 6 experimental groups were established as follows: A-group experimentally infected with *N*. *apis*; AH- group experimentally infected with *N*. *apis* and treated with cycloheximide; C-group experimentally infected with *N*. *ceranae*; CH-group experimentally infected with *N*. *ceranae* and treated with cycloheximide; T- Control, bees not infected with *Nosema* sp.; TH—Control, bees not infected with *Nosema* sp. but treated with cycloheximide.

**Table 1:** Experimental design. *A*. *mellifera iberiensis* bees were infected with *N*. *apis* or *N*. *ceranae* or non-infected.

	*N*. *apis* N = 25	*N*. *ceranae* N = 25	Uninfected N = 25
Cell cycle, mitochondria & hormone expression	Group A n = 10	Group C n = 10	Group T n = 10
Apoptosis expression*	Group AH n = 10	Group CH n = 10	Group TH n = 10
Apoptotic index*	Group AH n = 5	Group CH n = 5	Group TH n = 5

(*) Groups treated with cycloheximide on day 10 post infection.

### Gene expression study

The bees studied (Table 1) were killed by freezing in liquid nitrogen on day 11 post infection and they were stored at -80°C until use. The ventriculus (infection target tissue) and rectal ampoule (rectum) were individually separated by dissection of each bee and stored in different tubes. Ventriculi were used to study the gene expression and they were stored in RNAlater (Qiagen 76106). The ampoules were used to confirm *Nosema* infection in each bee or the uninfected state of the control bees.

The RNA from each ventriculus was extracted individually using the RNeasy Tissue Kit (Qiagen) following the Purification of Total RNA from Animal Tissues protocol with on-column DNase digestion (DNase, Qiagen GmbH). Reverse transcription of RNA to cDNA was performed using the QuantiTect RT Kit (Qiagen GmbH).

The candidate genes related with *A*. *mellifera* homeostasis (cell cycle, mitochondria activity, apoptosis and hormones; S1 Table) and the house-keeping (HK) genes were selected from the sequences available at GeneBank (http://www.ncbi.nlm.nih.gov/nuccore). Specific primers and TaqMan^®^ probes were designed using the Primer express (Applied Biosystems) software and the theoretical specificity was checked using the Basic Local Alignment Search Tool (BLAST) at GenBank (http://blast.ncbi.nlm.nih.gov/Blast.cgi). The efficiency of each reaction to study the gene regulation was determined by analyzing serial cDNA dilutions.

#### Selection of housekeeping genes

Four *A*. *mellifera* HK genes (*GAPDH*, *EF*, *β-Actin* and 18S; S1 Table) were evaluated to select the most appropriate ones for analysis [22]. To determine the stability of expression (M) of the selected reference genes in the honeybee tissues studied (ventriculi), the four HK genes were analyzed in duplicate in all the samples and the Ct values were subsequently determined with the geNorm software [23]. The two most stable genes (lowest M value) were *EF* and β-*Actin* and they were selected as the HK genes for the subsequent analysis of expression.

The relative expression for each gene was calculated using the Relative Expression Software Tools: REST MCS-version 2 (http://www.gene-quantification.de/download.html) and REST 2009-version 2.0.13 (Qiagen GmbH), which use the pair wise fixed reallocation randomization test [24, 25]. All the groups infected with either *N*. *apis* or *N*. *ceranae* (irrespective of the treatment with cycloheximide) were compared with the uninfected control groups using this software and the efficiency of every reaction was taken into account as recommended elsewhere [24]. The level of significance for determining the up or down regulation for each gene was also determined using this software.

#### Real-time quantitative PCR

All real-time quantitative PCR reactions were performed using the 7900 HT Sequence Detection System (Applied Biosystems). To assay expression, each 25 μl reaction contained 0.625 U of Taq (TaqMan Gold/Buffer A Pack, cod. 4304441, Applied Biosystems), 10 μl of diluted cDNA (1/100), 300 nM of each primer and 100 nM of each TaqMan probe. The PCR program involved an initial 2 minute incubation at 50°C, a 10 minute denaturation step at 95°C and 40 cycles of 15 seconds at 95°C and a 1 minute of annealing at 60°C. All the cDNA samples obtained after extraction from each bee were run in duplicate. Negative controls for DNA extraction, reverse-transcription and real-time PCR steps were also included. The Ct values were recorded for each gene studied in every sample and the average of the two replicates was calculated.

#### *Nosema* spp. infection checking

The success *of Nosema* infection was determined using real-time PCR in honey bee rectal ampoules, given that this part of the bee gut stores the microsporidia spores until their exit in the faeces. DNA was extracted from the ampoules that had been separated from the ventriculi using the DNeasy tissue protocol (Qiagen GmbH) and it was collected individually in microcentrifuge tubes (one bee per tube) before analyzing it by real-time PCR and TaqMan^®^ chemistry. The primers and probe sequences are shown in S2 Table.

#### Virus detection

In order to check the exposure of the bees to other naturally acquired and relevant pathogens, the presence of virus was also analysed in the cDNA (5 μL) from each bee in every group (A, AH, C, CH, T and TH). For each group, 10 μL of the mixture was analysed in duplicate for the presence of Kashmir bee virus (KBV), acute bee paralysis virus (ABPV), black queen cell virus (BQCV), chronic bee paralysis virus (CBPV), Deformed wing virus (DWV) and Israeli acute paralysis virus (IAPV) in duplicate. When a virus was detected in any group, all the bees from that group were individually analysed. The sequences of the primers and probes used are shown in S2 Table.

### Histology (TUNEL assay)

Five Bees from the cycloheximide treated groups (AH, CH and TH) were analysed histologically, to evaluate the effect of microsporidia infection in bees exposed to this strong inducer of apoptosis. The alimentary canal and other tissues (ventriculum with the Malpighian tubules attached, the small intestine and the rectum) were removed from the bees, divided into sections and fixed in 10% buffered formalin before they were paraffin embedded. The TUNEL (Terminal deoxynucleotide transferase mediated X-dUTP nick end labelling) assay was performed on 5 μm thick sections to quantify apoptosis, as described previously [26, 15]. Briefly, tissue sections were deparaffinised and rehydrated through an ethanol series before rinsing in phosphate buffered saline (PBS). The tissues were then treated with Proteinase K (20 μg ml–1, 15 min, room temperature) and then with 0.1% (w:v) Triton X-100 in PBS (10 min). Subsequently, the sections were incubated for 1 h at 37°C in the dark, using a humid chamber and the In situ Cell Death Detection Kit (Roche) according to the manufacturers’ instructions. The sections were then washed with PBS and mounted in Vectashield (Vector Labs) containing DAPI (Sigma) at a final concentration of 1 μg/ml, to label all the cell nuclei (blue). The sections were examined on an Olympus BX61 epifluorescence microscope equipped with filter sets for fluorescence microscopy: ultraviolet (UV, 365 nm, exciting filter UG-1). Photographs were obtained with a digital camera Olympus DP50 and processed using the Adobe PhotoShop 7.0 software (Adobe Systems). Uninfected bees and no treated with cycloheximide were used to determine the basal apoptosis (Basal control) just to show the natural apoptosis in the tissue. Cells undergoing apoptosis were scored (fluorescing green under blue excitation light) and the ventricular cells of three bees per group were counted to determine the number with apoptotic nuclei and the proportion of cells undergoing apoptosis (No. of apoptotic cells x 100 / total No. of cells) as described previously [15]. Approximately 100 cells were examined in representative areas of each ventricular sample (10 areas per sample, approximately 1,000 cells per ventriculum and bee).

## Results

The success of *Nosema* infection (checked in the bee ampoules) was reflected by the detection of spores in each group. Consequently, all the bees were successfully infected by either *N*. *ceranae* or *N*. *apis*, while no cross-infection between groups was detected and there was no infection in the uninfected groups. However, one sample from the group A and another bee from the group CH showed a very low level of infection (Ct value > 36 in *Nosema* infection analysis; see above) and they were not considered for the gene expression analysis.

In none of the samples were CBPV, ABPV, KBV, IAPV or DWV detected. Yet, as all the groups tested positive for BQCV, an additional analysis was carried out to determine the presence of the virus in individual bees. Since more than the 80% of the bees were positive for BQCV in all the groups, viral infection was not considered a significant factor and was not included in the subsequent analyses.

### Gene expression

The list of genes selected for study, and the primers and probes used are shown in S3 Table. All the primer pairs tested produced positive amplification with the exception of Cyclin H and BRUCE, thus these genes were not tested further.

#### Selection of reference HK genes

The ranking of the four candidate reference genes in the honey bee ventriculi according to their average expression stability (M value) was: *β-Actin* (0.060) <*EF* (0.066) <*GAPDH* (0.067) <*18S* (0.097), from the most stable (lowest M-value) to the least stable (highest M value). All the values were very low and they were much lower than the threshold limit of 1.5 recommended by GeNorm software, reflecting very stable expression. The pairwise variation (V) between the two or three normalization factors was 0 [22], indicating that the use of *β-Actin* and *EF* as HK were enough to perform all the subsequent analysis.

#### Apoptosis related genes

**Fig 1:**
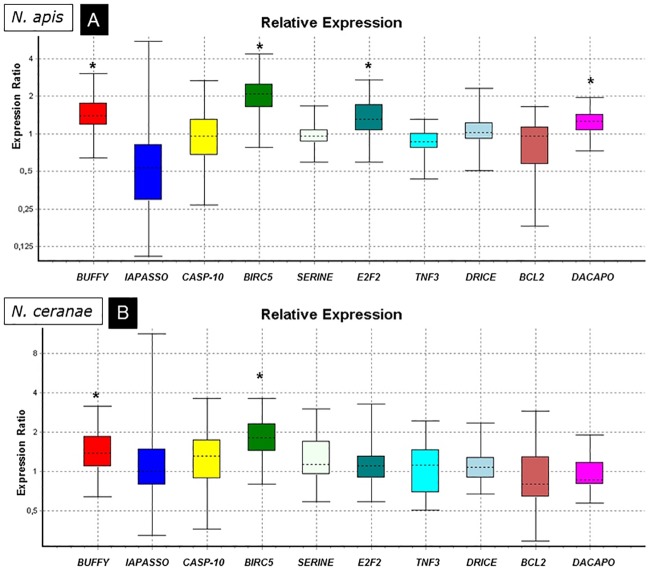
Relative expression ratio plots for apoptosis related genes. Analysis of groups treated with cycloheximide and infected with *N*. *apis* (A) or *N*. *ceranae* (B) relative to uninfected bees. (*) significant differences.

The expression of genes related to apoptosis (Fig 1) was analyzed in the honey bees infected either *N*. *apis* (AH; Fig 1A) or *N*. *ceranae* (CH, Fig 1B) and it was compared with that in the uninfected control group (TH), all of them treated with cycloheximide. The results showed the up-regulation of *Buffy* and *BIRC5* in both the AH and CH groups for the (P<0.05; Table 2) and the down-regulation of *IAPASSO* and *TNF3* in the AH group alone, that was close to the level of significance (P = 0.055 and P = 0.057, respectively). Finally, the *E2F* and *Dacapo* genes were up-regulated in the AH group (P<0.05).

**Table 2:** Expression of studied target genes.

		*N*. *apis*					*N*. *ceranae*				
Gene short ID	Reaction Efficiency	Expression	Std. Error	95% C.I.	P(H1)	Result	Expression	Std. Error	95% C.I.	P(H1)	Result
*EF*	0.969										
*Actin*	0.922										
*CYTOX*	0.921	0.829	0.557–1.253	0.420–1.828	0.174		1.208	0.905–1.629	0.715–2.094	0.059	
*TU-MITO*	0.773	0.860	0.594–1.307	0.447–1.878	0.262		1.096	0.788–1.455	0.610–1.789	0.375	
*S-12*	1.0	0.798	0.517–1.224	0.382–1.852	0.149		1.056	0.768–1.468	0.605–1.832	0.582	
*L16*	0.998	1.041	0.768–1.494	0.565–1.878	0.722		1.212	0.952–1.520	0.755–1.833	**0.022**	**UP**
*LSU*	0.917	0.778	0.458–1.326	0.279–2.860	0.219		1.145	0.878–1.524	0.670–1.805	0.155	
*M-PHASE*	0.8405	0.857	0.677–1.023	0.467–1.785	0.183		1.024	0.859–1.202	0.764–1.513	0.708	
*RING*	0.9535	0.777	0.437–1.349	0.358–2.752	0.221		0.580	0.342–1.005	0.282–1.803	**0.007**	**DOWN**
*B1 CYCLIN*	0.8715	1.393	1.071–1.871	0.856–2.324	**0.001**	**UP**	0.900	0.684–1.146	0.551–1.521	0.249	
*B3 CYCLIN*	0.9425	1.032	0.690–1.523	0.421–2.422	0.820		1.043	0.697–1.508	0.471–1.898	0.754	
*K CYCLIN*	0.804	0.92	0.685–1.230	0.541–1.629	0.434		1.011	0.795–1.288	0.646–1.595	0.896	
*E CYCLIN*	0.977	0.831	0.651–1.481	0.078–2.086	0.752		1.192	0.935–1.502	0.718–1.758	**0.047**	**UP**
*JH*	1.0	1.154	0.768–1.805	0.499–2.580	0.325		1.112	0.756–1.678	0.657–2.424	0.406	
*VG*	0.905	0.611	0.249–1.559	0.101–2.543	0.112		1.727	0.756–4.300	0.355–7.929	0.064	
*GAPDH*	0.9855	0.795	0.550–1.278	0.217–1.814	0.230		0.928	0.591–1.382	0.302–1.832	0.668	
*18S*	1.0	1.147	0.688–1.953	0.495–3.187	0.448		0.723	0.308–1.276	0.220–2.422	0.149	
*Buffy*	0.9175	1.422	1.052–1.945	0.783–2.495	**0.007**	**UP**	1.407	0.962–2.101	0.776–2.701	**0.034**	**UP**
*IAPASSO*	0.977	0.529	0.230–0.932	0.128–3.738	0.055		1.202	0.729–1.939	0.398–6.889	0.578	
*CASP-10*	0.994	0.951	0.572–1.677	0.348–2.439	0.800		1.256	0.779–2.109	0.434–3.180	0.264	
*BIRC5*	0.99	2.022	1.423–2.793	1.014–3.981	**0.000**	**UP**	1.834	1.273–2.704	0.842–3.375	**0.004**	**UP**
*SERINE*	0.9575	0.953	0.817–1.152	0.604–1.462	0.539		1.238	0.777–1.901	0.599–2.439	0.211	
*E2F2*	0.911	1.340	0.920–1.944	0.730–2.423	**0.040**	**UP**	1.158	0.836–1.502	0.648–2.823	0.337	
*TNF3*	0.867	0.840	0.648–1.040	0.469–1.201	0.057		1.041	0.607–1.667	0.522–2.259	0.806	
*DRICE*	0.946	1.041	0.787–1.354	0.530–1.867	0.721		1.102	0.804–1.454	0.699–2.035	0.382	
*BCL2*	0.988	0.790	0.471–1.207	0.320–1.417	0.174		0.881	0.587–1.522	0.324–2.468	0.529	
*Dacapo*	0.9545	1.229	0.976–1.523	0.775–1.815	**0.026**	**UP**	0.957	0.718–1.380	0.604–1.677	0.694	

EF and βActin were used as reference genes (2000 iterations). UP = up-regulation; DOWN = down-regulation. P(H1) = Probability of an alternate hypothesis that the difference between sample and control groups is due only to chance.

#### Cell cycle, mitochondrial activity and hormone related genes

**Fig 2:**
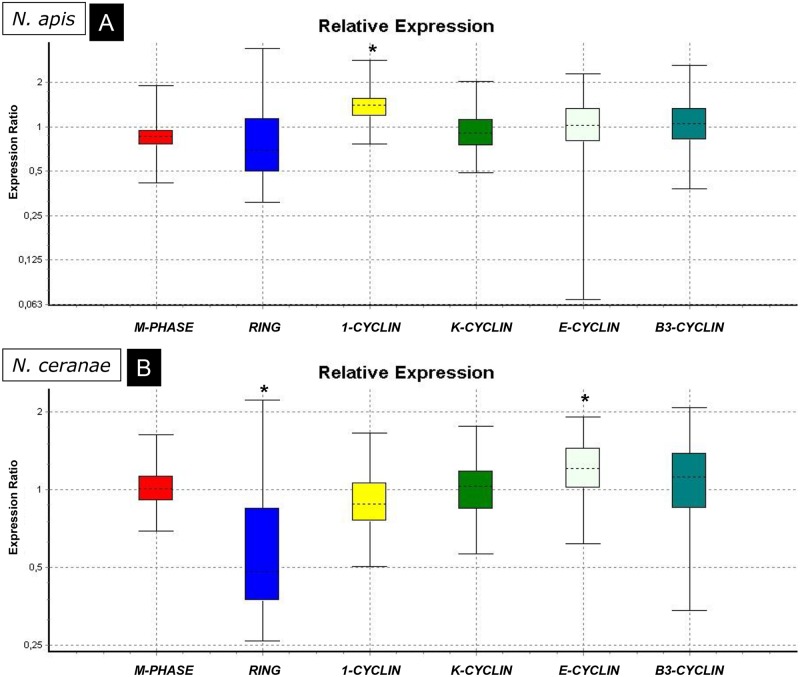
Relative expression ratio plots for genes related to the cell cycle. Analysis of the groups infected with *N*. *apis* (A) or *N*. *ceranae* (B) relative to the uninfected bees. (*) significant differences.

**Fig 3:**
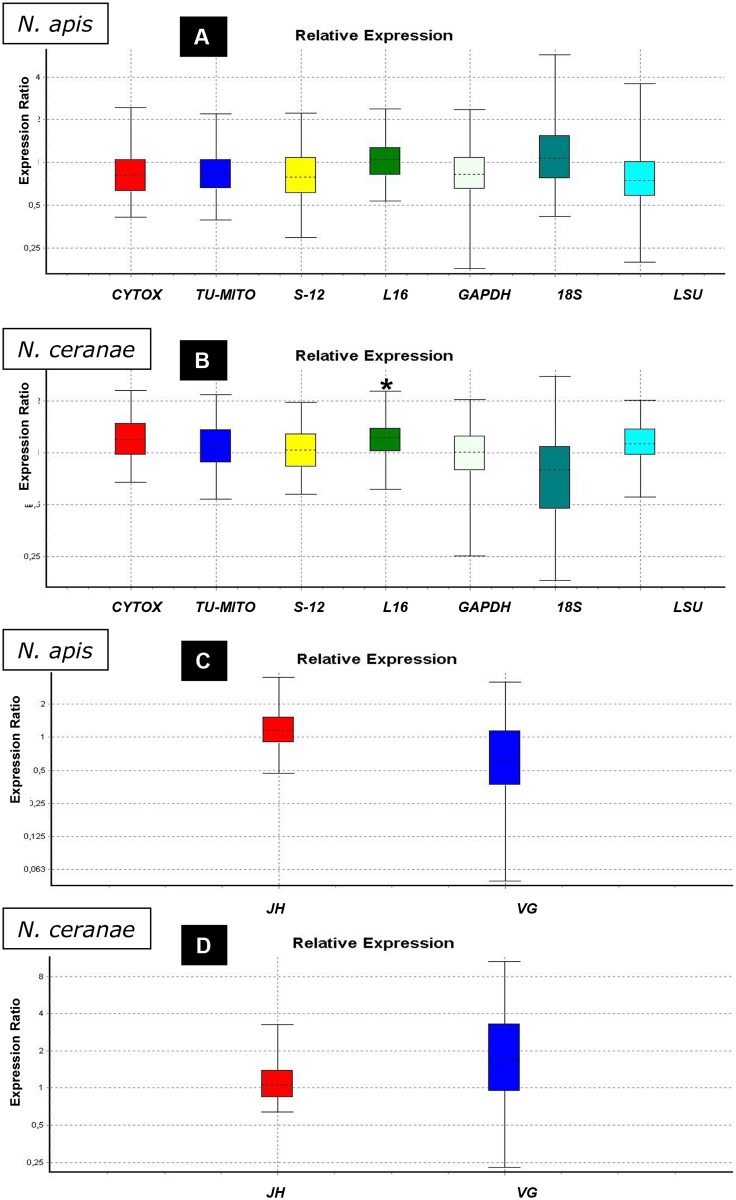
Relative expression ratio plots for genes related to mitochondrial and hormone activity. Analysis of groups infected with *N*. *apis* or *N*. *ceranae* relative to the uninfected bees. (*) significant differences. A) Mitochondrial activity in *N*. *apis* infected bees. B) Mitochondrial activity in *N*. *ceranae* infected bees. The expression for *GAPDH* and *18S* (not used as housekeeping genes) is also represented here. C) Hormone activity in *N*. *apis* infected bees. D) Hormone activity in *N*. *ceranae* infected bees.

The expression of the genes related to the cell cycle, mitochondrial activity and hormones (Fig 2) was studied in groups A (*N*. *apis* infected bees; Fig 2A) and C (*N*. *ceranae* infected bees, Fig 2B) and they were compared with those in group T (uninfected bees). Only *Cyclin B1* was up-regulated in group A (P<0.05; Table 2) while in the case of group C, one gene related to mitochondrial activity (*L16*) was up-regulated (P<0.05; Table 2) and another one (*CYTOX*) was very close to the level of significance for up-regulation (Fig 3A and 3B). Regarding cell cycle related genes (Fig 2), *RING* was down-regulated in group C while *Cyclin E* was up-regulated (both P<0.05; Table 2). No significant changes in the expression of *VG* or *JH* (Fig 3C and 3D) were observed in any of microsporidia infected bees, although it is important to note the up-regulation for *VG* close to the level of significance in group C.

### Histology (TUNEL assay)

**Fig 4:**
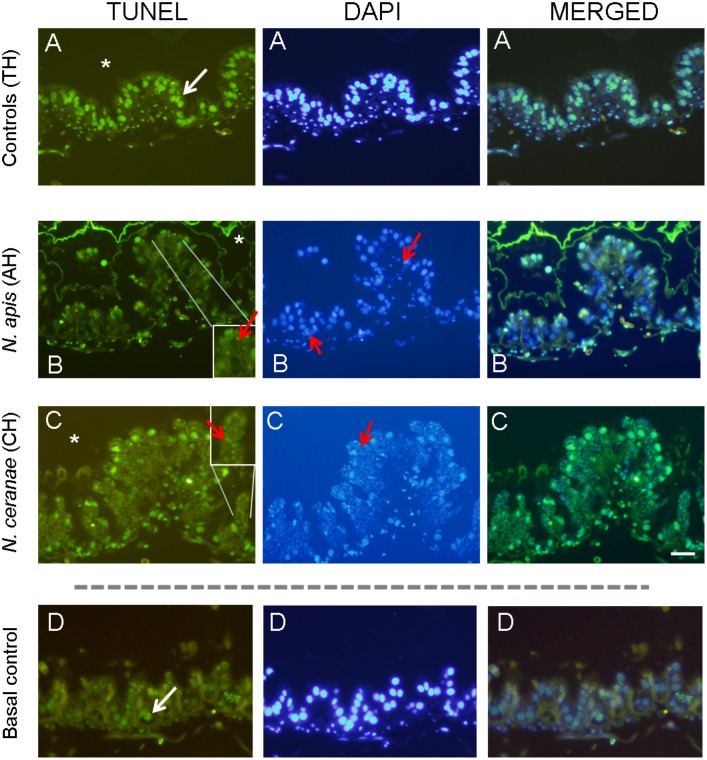
TUNEL assay. Representative TUNEL / DAPI stained and merged microscopic images of transverse sections from the ventriculum of A: uninfected (controls TH) B: infected with *N*. *apis* (AH) or C: *N*. *ceranae* (CH) honey bees and treated with cycloheximide. D: Basal apoptosis in uninfected bees and no treated with cycloheximide (Basal control) was very low. The ventriculum cells were counterstained with DAPI (blue). Scale bar = 100 μm. The ventricular lumen is indicated by an asterisk and spores inside ventricular cells are indicated with red arrows.

Our study showed that cycloheximide induced apoptosis in bees (Table 3; Fig 4) with the control bees (TH) showing a high level of cell death (69, 95% ± 14.35). Apoptosis appeared to be reduced in the microsporidia infected tissues although this was no significant (ANOVA, F = 1.109; P>0.05). This reduction in apoptosis was higher in *N*. *apis* infected ventriculi (27.86% reduction) than in the *N*. *ceranae* infected ventriculi (13.82% reduction). Apoptotic TUNEL positive cells (green stained) were detected along the epithelium in the ventriculi (Fig 4A, 4B and 4C). The amount of positive apoptotic cells were higher in the positive controls (TH, uninfected bees treated with cycloheximide; Fig 4A), compared to ventriculi of basal controls (uninfected, no cycloheximide treated bees, Fig 4D). Ventriculi from infected bees either infected by *N*. *apis* (Fig 4B) or *N*. *ceranae* (Fig 4C) showed a lower amount of TUNEL positive cells compared to ventriculi from TH group bees (Fig 4A).

**Table 3:** Percentage of ventricular cell nuclei undergoing apoptosis in cycloheximide treated bees.

		Average	Std. deviation	% Reduction in Apoptosis
Controls (uninfected)	TH	69.95	14.35	-
Infected with *N*. *apis*	AH	50.46	11.70	27.86%
Infected with *N*. *ceranae*	CH	60.27	13.72	13.82%

## Discussion

The natural response of an infected cell is to undergo apoptosis in order to prevent the multiplication and dissemination of the invader. Conversely, the invader must find ways to evade this to be able to reproduce [6]. In this work, microsporidia have been shown to successfully manipulate the host cell’s metabolism to progress along the parasite’s life cycle, not only modifying the expression of apoptotic genes but also other important routes implicated in the host’s cell cycle. These responses seem to offer an important advantage, because similar conclusions have been reported for many intracellular parasites [3–7, 14].

To investigate the expression of some genes related to apoptosis, a potent intrinsic inducer of apoptosis was used (cycloheximide, a protein synthesis inhibitor that halts translational elongation [27]). Studies of the apoptotic index derived when using the TUNEL assay and caspase-3 staining suggested that *N*. *ceranae* could prevent the epithelial cells of infected honey bee tissue (ventriculi) from undergoing apoptosis [15]. For that reason, in this work a high level of apoptosis was induced in a group of bees in order to determine if *N*. *ceranae* or *N*. *apis* infection blocked the effect of this strong apoptosis inducer. The tissue of uninfected-bees treated with cycloheximide exhibited more apoptosis than that infected by microsporidia, with a clear reduction in the apoptotic index evident in TUNEL assays. Indeed, this is the first study to confirm this effect in *N*. *apis* infected tissues. As such, microsporidia infection clearly inhibits cycloheximide-induced apoptosis (intrinsic route) as described in *Leishmania donovani* infected macrophages [28].

**Fig 5:**
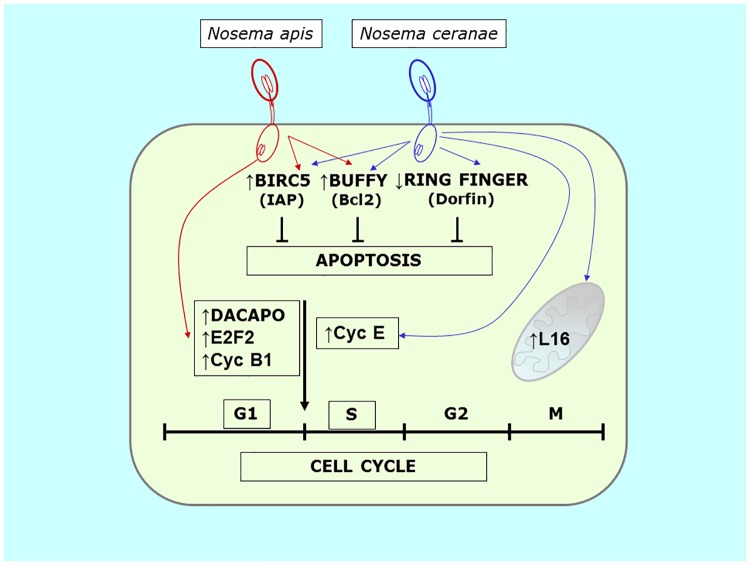
Scheme of the genes altered in *N*. *ceranae* and *N*. *apis* infection in honey bees and the effects on host’s cell cycle, apoptosis and mitochondrial activity.

This inhibition of apoptosis is consistent with the observed modifications in the expression of the apoptosis related genes studied. Infection with both microsporidia species up-regulates *buffy* and *BIRC5*, two genes with important function in programmed cell death (Fig 5). The *BIRC5* (baculoviral IAP repeat-containing 5; also called Survivin) is a member of the inhibitor of apoptosis (IAP) family and it encodes a protein that inhibits caspase activation, thereby negatively regulating apoptosis [29–31]. On the other hand, *buffy* was identified in *Drosophila melanogaster* and it encodes a Bcl-2-like pro-survival protein [32]. The Bcl-2 (B cell lymphoma-2) protein family plays a central role in the intrinsic apoptotic pathway, controlling the integrity of the outer mitochondrial membrane. Consequently, in honey bees the up-regulation of *BIRC5* and *buffy* is associated with a reduction in the number of cells that finally enter apoptosis in the infected tissues. These results agree with a previous work where inhibitors of apoptosis proteins were seen to be up-regulated after *N*. *ceranae* infection in sensitive bees while c*aspase 10* gene expression was not modified [16]. Consequently, IAP family seems to play a key role in the inhibition of these processes. Additionally, this is the first report of such changes in *N*. *apis* infected bees, confirming that the inhibition of apoptosis is a common response of the host to the benefit of this group of intracellular parasitic fungi.

We also note that *IAPASSO* (viral IAP-associated factor; VIAF) and *TNF3* (Tumor Necrosis Factor receptor-associated factor 3 interacting protein 1) displayed a tendency towards down-regulation (P = 0.055 and p = 0.057, respectively) in *N*. *apis* infected bees. IAPASSO is a conserved inhibitor of apoptosis interacting factor that modulates caspase activation during apoptosis [33] and TNF3 is a member of the TNF superfamily proteins involved in complex pathways that regulate cellular survival, proliferation, differentiation and apoptosis [34, 35]. Consequently, the tendency to down-regulate both these genes in conjunction with *buffy* and *BIRC5* overexpression fits into the theory that apoptosis is inhibited by the microsporidium *N*. *apis*.

Regarding the cell cycle, it has been suggested that the G1 phase is vital to decide if a cell will go on to differentiate, multiply, apoptose or become quiescent or senescent [36]. A balance between cell proliferation and apoptosis is essential for the development of the multicellular organism [32]. In our study, a modification of cell cycle related genes was observed in an epithelium (bee ventriculi) considered to be non-replicative beyond its basal germinative layer.

Curiously, while both microsporidia inhibit apoptosis through the same pathways (*Buffy* and *BIRC5*), the modifications to cell cycle related genes differ. However, both *Nosema* species caused the up-regulation of some cyclins in the infected cells: over expression of *cyclin B1* (*cycB1*) by *N*. *apis* and *cyclin E* (*cycE*) by *N*. *ceranae* (Fig 5).

*N*. *apis* infected tissues also over expressed *dacapo* and *E2F2*, both of them closely related with the progression of the G1-S phase. Dacapo is a cyclin dependent kinase inhibitor that coordinates the rates of G1-S and G2-M progression, maintaining normal rates of proliferation when cell cycle controls are perturbed [37]. Also, E2F2 (E2F Transcription Factor 2) is a transcription factor maximally expressed late in G1 that plays a pivotal role in G1/S transition [38, 39]. Over expression of both these genes suggest that the infected cells undergo the G1-S phase progression. However, the up-regulation of *cyclin B1* (G2/Mitotic-specific Cyclin-B1) was initially unexpected since it codes for a regulatory protein involved in mitosis and it contributes to all or none switch-like behavior of the cell in deciding to commit to mitosis. *E2F2* was overexpressed in rabbit corneal endothelial cells (arrested in G1-phase) and it coexisted with high levels of cyclin B1 [40] and there was strong evidence of progression from the G1-phase arrested state to at least the G2-phase in these tissues. Our data suggest a progression from G1 to S phase is being promoted in *N*. *apis* infected tissues (Fig 5).

In *N*. *ceranae* infected bees, significant changes were observed in the expression of *cyclin E* (*CycE*) and *RING* (RING-finger protein 19 or *Dorfin*). A previous study of the transcriptome of *A*. *mellifera* infected by *N*. *ceranae* identified mRNA for a number of cyclins (as cyclin E) and other proteins closely related to cell cycle [41] although no data on expression were available. Our results show an up-regulation of *CycE*, which appears to be the most important cyclin for the G1 to S transition [42, 43]. Consequently, and as in *N*. *apis* infection, G1-S phase progression is apparently being stimulated in *N*. *ceranae* infected cells, albeit through this different pathway.

Additionally, down regulation of the *RING* gene (RING-finger protein 19 or *Dorfin*) was observed after *N*. *ceranae* infection. This gene codes for the E3 ubiquitin-protein ligase RNF19A which in *Caenorhabditis elegans* has been described as one of the three core components of a complex that target toxins and intracellular pathogen proteins for degradation [44]. In general these RING finger E3 proteins can influence the balance between proliferation and apoptosis. In response to apoptotic stimuli the E3 activity of IAPs leads to their auto-ubiquitination, degradation, and progression toward cell death [45]. Ubiquitin pathways have been described as a specific mechanism of host defence against microsporidia infection [44]. Although *RING* was studied in bees from Group C here (cell cycle assay) its down-regulation provides more information about apoptosis and corroborates the results obtained in group CH (treated with cycloheximide). In fact, these data suggest another pathway to inhibit apoptosis that may be activated by *N*. *ceranae* infection.

Microsporidia are obligate intracellular parasites with extremely reduced genomes and a dependence on host-derived ATP. Honey bee cells infected with *N*. *ceranae* were enlarged and the cytoplasm contained a larger number of host mitochondria and free ribosomes. Several mitochondria were close to and surrounded the plasmalemma of meronts [18], similar to sporulating *Buxtehucdea scaniae* [46], suggesting a energy supply external to the parasite. For this reason, the regulation of five host mitochondrial related genes was also included in this study. However only one of them (*MRP L16*) was seen to be up-regulated and only in *N*. *ceranae* infected bees (Fig 5). *MRP L16* is a nuclear gene that encodes for the mitochondrial ribosomal protein L16. This polypeptide plays an important role in the assembly and structure of the peptidyl transferase centre of the ribosome, and it is crucial for the correct behavior of mito-ribosomes in yeast [47]. MRP over expression depends mainly on glucose repression and de-repression, which decreases or elevates MRP mRNA and protein levels, respectively [40]. It is important to note that *N*. *ceranae* infection has been reported to produce a nutritional and energetic stress in bees that leads to a major ingestion of food that is rich in glucose [48–50]. On the other hand, the stable accumulation of L16 depends on the presence of mitochondrial rRNA [47], so changes in the integrity of mitochondrial activity directly affect the levels of this protein too. The related genes studied in this work, *CYTOX*, *TU-MITO*, *S12* and *LSU*, suggest normal mitochondrial activity that would allow the accumulation of L16 protein inside this organelle. In fact, the expression of Cytochrome C oxidase subunit VI gene is remarkable high, at levels that are almost significant(p = 0.059), which would fit into the theory of the mitochondria overworking during *N*. *ceranae* infection. Mitochondrial protein modulation by intracellular parasites has been reported previously in *Toxoplasma gondii* infection [1]. The direct or indirect perturbation of mitochondria dynamics may play a crucial role in the sustained viability of host cells preserving the pathogen’s replication niche or alternatively triggering the apoptotic process to circumvent immune effector cells. It will be crucial to identify the host’s molecular mechanisms and the pathogenic factors involved in such control [7]. Finally, no significant results were observed in terms of mitochondrial activity in bees infected by *N*. *apis*. This again indicates that both microsporidia affect the same host in a different manner.

Finally, the expression of vitellogenin and juvenile hormone were also assessed here. These molecules have been related to the bees’ immune response and to other physiological activities such as in behavioural development [51, 52], and some modifications to their expression were previously reported in bees infected by *Nosema* spp. [53–55]. However, no significant modification was detected here for either hormone, probably because the effects of infection were only studied in the target tissue (ventricular cells) and the hormone secreting organs were not assessed.

In multicellular organisms, cell proliferation and death must be regulated to maintain tissue homeostasis. Many observations suggest that this regulation may be at least partially achieved by coupling the process of cell cycle progression and programmed cell death through a set of common factors. Evidence is accumulating that manipulation of the cell cycle may either prevent or induce an apoptotic response arguing in favor of a link between the cell cycle and apoptosis [56]. For bee microsporidia, this is the first report describing molecular pathways related to parasite-host interactions that probably serve to promote their own survival within the cell. New routes of apoptosis involving *Buffy* and *BIRC5* were seen to be modified by both microsporidia, while the effects of the microsporidia on the expression of genes related with the cell cycle indicate that both *Nosema* species apparently promote the G1/S phase through different pathways (c*yclin B1*/ *dacapo*/ *E2F2* by *N*. *apis*, c*yclin E* by*N*. *ceranae*). Lastly, the changes to mitochondrial markers appear to further support a distinct response to the two *Nosema* species studied as only *N*. *ceranae* infection leads to higher mitochondrial activity. All these evidences demonstrate the strong interaction between the host cells and this group of parasites. These results increase further our knowledge on the specific pathways that these microsporidia uses to survive and multiply in the host, shedding light on the pathogenic mechanism of *A*. *mellifera* microsporidia, and hence on the relationship between these insects and their intracellular parasites.

## Supporting information

S1 Table List of genes selected from *A*. *mellifera*. Genes related to cell cycle, mitochondrial activity, apoptosis, hormone activity and housekeeping genes.
(DOCX)

S2 Table List of Primers and probes to study honey bee pathogens. Sequences of primers and probes used in Real-Time PCR for the detection of pathogens in individual bee samples and PCR conditions were obtained from previously published works.
(DOCX)

S3 Table List of primers and probes designed for *A*. *mellifera*. Sequences designed for Real-Time PCR to study the cell cycle, mitochondrial activity, apoptosis, hormone activity and housekeeping genes. All of them were designed in this study except 18S and EF that have been published previously.
(DOCX)
