# Supplementary material for: Microsporidia infection impacts the host cell's cycle and reduces host cell apoptosis
Source: PLoS One. 2017 Feb 2;12(2):e0170183. doi: 10.1371/journal.pone.0170183 (PMC5289437; doi:10.1371/journal.pone.0170183)
Supplement: S1 Table — Genes related to cell cycle, mitochondrial activity, apoptosis, hormone activity and housekeeping genes. (DOCX) [file pone.0170183.s001.docx]

**S1 Table.** **List of genes selected from *A. mellifera*.** Genes related to cell cycle, mitochondrial activity, apoptosis, hormone activity and housekeeping genes.

| GEN NAME | Short Name (Short ID) | GeneBank ID | Reference |
| --- | --- | --- | --- |
| **CELL CYCLE RELATED GENES** |  |  |  |
| Cyclin H | *H CYCLIN* | XM_393939.3. PREDICTED: *Apis mellifera* similar to Cyclin H |  |
| M-phase inducer phosphatase | *M-PHASE* | XM_001122242.1. PREDICTED: *Apis mellifera* similar to M-phase inducer phosphatase (Cdc25-like protein) |  |
| RING finger protein 19 (Dorfin) | *RING* | XM_623725.2. PREDICTED: *Apis mellifera* similar to RING finger protein 19 (Dorfin) |  |
| G2/mitotic-specific Cyclin B | *B1 Cyclin* | XM_624168.2. PREDICTED: *Apis mellifera* similar to G2/mitotic-specific Cyclin-B1 |  |
| Cyclin K | *K Cyclin* | XM_394536.1. PREDICTED: *Apis mellifera* similar to Cyclin K |  |
| G1/S-specific Cyclin E | *E Cyclin* | XM_394802.1. PREDICTED: *Apis mellifera* similar to G1/S-specific Cyclin-E |  |
| Cyclin B3 | *B3 Cyclin* | XM_397108.2. PREDICTED: *Apis mellifera* similar to CycB3 |  |
|  |  |  |  |
| **MITOCHONDRIA ACTIVITY** |  |  |  |
| Mitochondrial ribosomal protein L16 | *L16* | NM_001185104.1. *Apis mellifera* mitochondrial ribosomal protein L16 (mRpL16) |  |
| Cytochrome c oxidase subunit VIa | *CYTOX* | GU358185.1. *Apis mellifera* clone AMDH2786 cytochrome c oxidase subunit VIa mRNA |  |
| Elongation factor Tu mitochondrial | *TU-MITO* | XM_623048.2. PREDICTED: *Apis mellifera* similar to Elongation factor Tu mitochondrial |  |
| 40S ribosomal protein S12 | *S-12* | XM_393221.3. PREDICTED: *Apis mellifera* similar to 40S ribosomal protein S12 |  |
| Mitochondrial ribosomal large subunit | *LSU* | X05011.1. Honeybee mitochondrial large ribosomal RNA |  |
|  |  |  |  |
| **APOPTOSIS** |  |  |  |
| Bcl-2 inhibitor of transcription | *BCL2* | XM_395591.2. PREDICTED: *Apis mellifera* similar to Bcl-2 inhibitor of transcription |  |
| Buffy (similar to *Drosophila* Bcl-2-like protein) | *Buffy* | XM_395083.3. PREDICTED: *Apis mellifera* similar to Buffy |  |
| viral IAP-associated factor | *IAPASSO* | XM_394510.3. PREDICTED: *Apis mellifera* similar to viral IAP-associated factor |  |
| Baculoviral inhibitor of apoptosis repeat-containing Birc6 | *BRUCE* | XM_394589.3. PREDICTED: *Apis mellifera* similar to Bruce (Bruce/APOLLON) |  |
| Caspase 10 | *CASP-10* | XM_001120830.1. PREDICTED: *Apis mellifera* similar to caspase 10 |  |
| Serine-protein kinase ATM | *SERINE* | XM_001121440.1. PREDICTED: *Apis mellifera* similar to Serine-protein kinase ATM |  |
| Baculoviral IAP repeat-containing 5 | *BIRC5* | XM_392920.3. PREDICTED: *Apis mellifera* similar to baculoviral IAP repeat-containing 5 |  |
| TNF receptor-associated factor 3 | *TNF3* | XM_623859.2. PREDICTED: *Apis mellifera* similar to TNF receptor-associated factor 3 interacting protein 1 |  |
| Caspase precursor (drICE) | *DRICE* | XM_395697.3. PREDICTED: *Apis mellifera* similar to Caspase precursor (drICE) (LOC412235), mRNA |  |
| Dacapo (C[yclin dependent kinase inhibitor](http://www.sdbonline.org/fly/aimain/6signal.htm)) | *Dacapo* | XM_001121044.1. PREDICTED: *Apis mellifera* similar to dacapo |  |
| E2F transcription factor 2 | *E2F2* | XM_396223.2. PREDICTED: *Apis mellifera* transcription factor E2F2-like |  |
|  |  |  |  |
| **OTHER GENES (HORMONE ACTIVITY)** |  |  |  |
| Vitellogenin precursor | *VG* | AJ517411.1. *Apis mellifera* mRNA for vitellogenin precursor |  |
| Juvenile hormone-inducible protein 26 | *JH* | XM_001122394.1. PREDICTED: *Apis mellifera* similar to Juvenile hormone-inducible protein |  |
|  |  |  |  |
| **HOUSEKEEPING** |  |  |  |
| Elongation Factor | *EF* |  | [3] |
| 18S rDNA | *18S* |  | [2] |
| Glyceraldehyde 3 phosphate dehydrogenase 1 | *GAPDH* | 1. XM_393605.4. PREDICTED: *Apis mellifera* glyceraldehyde 3-phosphate dehydrogenase |  |
| β-Actin | *Actin* | AB023025.1. *Apis mellifera* mRNA for actin |  |
|  |  |  |  |

2. Ward LI, Waite R, Boonham N, Fisher T, Pescod K, Thompson H, et al. First detection of Kashmir bee virus in the UK using real-time PCR. Apidologie 2007;38: 181-190.

3. Budge GE, Pietravalle S, Brown M, Laurenson L, Jones B, Tomkies V, Delaplane KS. Pathogens as predictors of colony strength in England and Wales. PLoS ONE 2015;10(7):e0133228.
