# Supplementary material for: Microsporidia infection impacts the host cell's cycle and reduces host cell apoptosis
Source: PLoS One. 2017 Feb 2;12(2):e0170183. doi: 10.1371/journal.pone.0170183 (PMC5289437; doi:10.1371/journal.pone.0170183)
Supplement: S2 Table — Sequences of primers and probes used in Real-Time PCR for the detection of pathogens in individual bee samples and PCR conditions were obtained from previously published works. (DOCX) [file pone.0170183.s002.docx]

**S2 Table.** **List of Primers and probes to study honey bee pathogens**. Sequences of primers and probes used in Real-Time PCR for the detection of pathogens in individual bee samples and PCR conditions were obtained from previously published works.

| **Name** | **FORWARD (5′ - 3′)** | **REVERSE (5′ - 3′)** | **PROBE (5’-FAM - 3’-TAMRA)** | **Reference** |
| --- | --- | --- | --- | --- |
| BQCV | GGTGCGGGAGATGATATGGA | GCCGTCTGAGATGCATGAATAC | TTTCCATCTTTATCGGTACGCCGCC | [4] |
| KBV | ACCAGGAAGTATTCCCATGGTAAG | TGGAGCTATGGTTCCGTTCAG | CCGCAGATAACTTAGGACCAGATCAATCACA | [4] |
| IAPV | RCRTCAGTCGTCTTCCAGGT | CGAACTTGGTGACTTGARGG | TTGCGGCAATCCAGCCGTGAAAC | [5] |
| DWV | CCTGGACAAGGTCTCGGTAGAA | ATTCAGGACCCCACCCAAAT | CATGCTCGAGGATTGGGTCGTCGT | [4] |
| APV | TCCTATATCGACGACGAAAGACAA | GCGCTTTAATTCCATCCAATTGA | TTTCCCCGGACTTGAC | [4] |
| CPV | TCTGGCTCTGTCTTCGCAAA | GATACCGTCGTCACCCTCATG | TGCCCACCAATAGTTGGCAGTCTGC | [4] |
| *N. apis* | ATTTACACACCAGGTTGATTCTGC | TGAGCAGTCCATCTTTCAGTACATAGT | TGACGTAGACGCTATTC (MGB) | [3] |
| *N. ceranae* | TTGAGAGAACGGTTTTTTGTTTGAG | TTCCTACACTGATTGTGTCTGTCTTTAA | ATAATAGTGGTGCATGGCCGTTTTCAATGG | [3] |

3. Budge GE, Pietravalle S, Brown M, Laurenson L, Jones B, Tomkies V, Delaplane KS. Pathogens as predictors of colony strength in England and Wales. PLoS ONE 2015;10(7):e0133228.

4. Chantawannakul P, Ward L, Boonham N, Brown M A. Scientific Note on the Detection of Honeybee Viruses Using real-time PCR (TaqMan) in Varroa Mites Collected from a Thai Honeybee (*Apis mellifera*) Apiary. J Invertebr Pathol. 2006;91: 69-73.

5. Kajobe R, Marris G, Budge G, Laurenson L, Cordoni G, Jones B, et al. First molecular detection of a viral pathogen in Ugandan honey bees. J. Invert. Pathol. 2010;104: 153–156.
