# Supplementary material for: Microsporidia infection impacts the host cell's cycle and reduces host cell apoptosis
Source: PLoS One. 2017 Feb 2;12(2):e0170183. doi: 10.1371/journal.pone.0170183 (PMC5289437; doi:10.1371/journal.pone.0170183)
Supplement: S3 Table — Sequences designed for Real-Time PCR to study the cell cycle, mitochondrial activity, apoptosis, hormone activity and housekeeping genes. All of them were designed in this study except 18S and EF that have been published previously. (DOCX) [file pone.0170183.s003.docx]

**S3 Table. List of primers and probes designed for *Apis mellifera*.** Sequences designed for Real-Time PCR to study the cell cycle, mitochondrial activity, apoptosis, hormone activity and housekeeping genes. All of them were designed in this study except 18S and EF that were previously published as indicated.

| **GEN SHORT NAME** | | **FORWARD** | | **REVERSE** | | | | **Probe (TaqMan®)** | **Reference** |
| --- | --- | --- | --- | --- | --- | --- | --- | --- | --- |
| **CELL CYCLE RELATED GENES** | | | | | | | | |  |
| *H Cyclin* | | XM_393939-268F ATTTCTGTCGTCGTTTTACTCCTT | | XM_393939-358R CGCTGTTTCTGAGATAGAATCTTTTG | | | | XM_393939-296TTGCCACGTGCTACTATAGCAACTGCATTACATTAT | This work |
| *M-PHASE* | | XM_001122242-333FGTCAAAACGAGACAGGTTTCGAT | | XM_001122242-401RCGGCATTCGGACATTCG | | | | XM_001122242-357TCGTGGAGCCTCTTGCTGTTGCTCC | This work |
| *RING* | | XM_623725.2-2078FCCGAGCGTGTCCGATTTG | | XM_623725.2-2150RCCACACGTGCTGACAATTCC | | | | XM_623725.2-2099TATCATGTCAGTTTCATCGAGGCGGATCA | This work |
| *B1 Cyclin* | | XM_624168-1113FGAAGTACGCCCAATCGAAATG | | XM_624168-1194RCGCAGTGGATATAGCAAATATCGT | | | | XM_624168-1135TATGAAGATTAGCACACGACCGGAACTT | This work |
| *K Cyclin* | | XM_394536-1072FCCAATGCATCATATGGGTTCCT | | XM_394536-1149RTTGAAATGGTGCCGGTGTT | | | | XM_394536-1098TTACCACTAGACCTGCACCACCTGCTCCT | This work |
| *E Cyclin* | | XM_394802-383FTGGGAGATCAAAAGACTATTACTCAAAG | | XM_394802-463RTTGCTCGCATTCTTGGTTGTA | | | | XM_394802-412TAATCCACAAATGTTTCAAAGGCACCCAA | This work |
| *B3 Cyclin* | | XM_397108-176FAAAGATCTGCATTGGGAAATATTACC | | XM_397108-260RTTTACTGCTTTCTTTGGTTCTTGTGT | | | | XM_397108-203TATGCAATTGGAAAAACATTGGGTACACATCA | This work |
|  |  |  | |  | | | |  | This work |
| **MITOCHONDRIA ACTIVITY** | | | | | | | | |  |
| *L16* | | NM_001185104-228FTGCGTGGTCCAGAATTATTTCA | | NM_001185104-302RTTCTACCACCACCAGTCGCTATT | | | | NM_001185104-251TAATACTCTTTTGCATAAACAGTACGGCA | This work |
| *CYTOX* | | GU358185-6FTGCAGGAACTGCTGTACTCATAAAA | | GU358185-85RTCCATACCTCGCGTGGAAGT | | | | GU358185-34TCGAGGATATTAAAGGTAGGCCAATTTGGCC | This work |
| *TU-MITO* | | XM_623048-151FCGCAATTCGTCCGATAGTTTGT | | XM_623048-216RTTTCAACACGAAATCTTCGAACA | | | | XM_623048-174TCGCGAGCGTCGCCCTCATC | This work |
| *S-12* | | XM_393221-262FAAGAGATTGCATGAGAAAGGATCA | | XM_393221-343RCGCCTTTTGCGAATGGTTT | | | | XM_393221-287TACAAGAAGCAAAAGAAGAAGAAAAATCCCTTAAGTGG | This work |
| *LSU* | | X05011-1012FTTAGGGATAACAGCGTAATATCTTTTGA | | X05011-1106RACTACTGCGCCTAATTTTCATCTTAAT | | | | X05011-1041TAGACCATATAGATAAAGATGTTTGCGACCTCGATG | This work |
|  |  |  |  | | | |  | |  |
| **APOPTOSIS** | | | | | | | | |  |
| *BCL2* | | XM_395591-289FGGACTTTTGACAAATATAATACAAGATGCT | | XM_395591-379RCAGCTGGACCAGGACCAATT | | | | XM_395591-321TATTTACCCAAGTGAAACCAGGAAGTAGAACAGTATGTG | This work |
| *Buffy* | | XM_395083-41FCATTGCCGATGCCTGAAAA | | XM_395083-113RCCGACGAATCCAGACATTGA | | | | XM_395083-61TTCTGAATGGCAGGAATTCTCCACGGA | This work |
| *IAPASSO* | | XM_394510-661FGGACCAATAGAATTAAGAGGCATGA | | XM_394510-751RTGGTAATTTTTGTTGGTATAGCTTCTG | | | | XM_394510-687TATTAACTGAAGCAGAACTGGAATGGATGTTAGGACA | This work |
| *BRUCE* | | XM_394589-11867FCCCCGTGCGCGTCTT | | XM_394589-11985RCTGGGCTCTCTTCCGATCTCT | | | | XM_394589-11885TCAGCCGCTTACGTCCCTCCCCA | This work |
| *CASP-10* | | XM_001120830-632FGGGAAAAAAAAGGAGTACTAAATGGAATA | | XM_001120830-725RTCTGGAAATTGAAAATCGGAAGA | | | | XM_001120830-666TCGTTGCTCAATTACCCGAAATAAAGTCATATCGA | This work |
| *SERINE* | | XM_001121440-2041FTGTTGTACTCATATGCATCCAGTGA | | XM_001121440-2144RGCTATGCTGCGGGTATATGCT | | | | XM_001121440-2093TCATCACCTGAAACATGGTTTGAACGCAG | This work |
| *BIRC5* | | XM_392920-306FCTTCTGACAATTCGTGCAATCC | | XM_392920-379RGGGTTCTTTCTTACCACCCACTAC | | | | XM_392920-330TAGCGGATGGCTGCTGCTGGTTTT | This work |
| *TNF3* | | XM_623859-932FCTAAACCAAGAACTTTTTTACGTCCAT | | XM_623859-1054RTTCCCATAGGTGTTAGTATCTCTTCATTAA | | | | XM_623859-980TCTAGACCTGCAGCACCTAAAA TTCGAGGAAAA | This work |
| *DRICE* | | XM_395697-155FTTGGAGATGCATTTGGATGTTC | | XM_395697-231RACGGTCCGTAGGTGCTATTTGT | | | | XM_395697-178TAGAAACACTGTCAGTCCTCTTATGGGCCCT | This work |
| *Dacapo* | | XM_001121044-110FTATTCGGACCCGTCGATCAC | | XM_001121044-176RATGGATTGAGCGCGAAGCT | | | | XM_001121044-134TCGGCTCGTGCATTGGCGGA | This work |
| *E2F2* | | XM_396223-1516FCGCGCTTTTGTGTGAATCC | | XM_396223-1581RTCCGTTTGGAGTTGGAATTTG | | | | XM_396223-1536TATGATTATGGACCAATGGGTGGTG | This work |
|  | |  | |  | |  | |  |  |
| **OTHER GENES (HORMONE ACTIVITY)** | | |  | |  | | |  |  |
| *VG* | | AJ517411-2857FTTGACCAAGACAAGCGGAACT | | AJ517411-2928RAAGGTTCGAATTAACGATGAAAGC | | | | AJ517411-2879TTCCAGGCGCAGATCAATCCCGA | This work |
| *JH* | | XM_001122394-372FGATGGCCGCGTTTCGA | | XM_001122394-434RCGCCTCTCCTTCGTCACGTA | | | | XM_001122394-389TAGATCGCGAGATTCCACGCAAAAGC | This work |
|  | |  | |  | |  | |  |  |
| **HOUSEKEEPING GENES** | | |  | |  | | |  |  |
| *EF* | | EF1 FCTGGTACCTCTCAGGCTGATTGT | | EF1 RGCATGCTCACGAGTTTGTCCATTCT | | | | EF1 TTGCTTCGAACTCTCTCCAGTACCAGCAGCAACA | [1] |
| *Actin* | | AB023025-205F GTATGCCAACACTGTCCTTTCTG | | AB023025-364R AAGAATTGACCCACCAATCCA | | | | AB023025-283T CCTAGCACCATCCACCATGAAAATTAAGATCATC | This work |
| *GAPDH* | | XM_393605-74F CTGATGCACCCATGTTTGTTTG | | XM_393605-281R AATTTTGCAGAAGGTGCATCAAC | | | | XM_393605-213T AGGTCTTATGACTACTGTTCATGCTGTTACTGCTACACA | This work |
| *18S* | | AJ307465-955FTGTTTTCCCTGGCCGAAAG | | AJ307465-1016RCCCCAATCCCTAGCACG AA | | | | AJ307465-975TCCCGGGTAACCCGCTGAACCTC | [2] |

1. Martin SJ, Highfield AC, Brettell L, Villalobos EM, Budge GE, Powell M, et al. Global honey bee viral landscape altered by a parasitic mite. Science 2012;336: 1304–1306.

2. Ward LI, Waite R, Boonham N, Fisher T, Pescod K, Thompson H, et al. First detection of Kashmir bee virus in the UK using real-time PCR. Apidologie 2007;38: 181-190.
